# Supplementary material for: An investigation into the effectiveness of using acoustic touch to assist people who are blind
Source: PLoS One. 2023 Oct 25;18(10):e0290431. doi: 10.1371/journal.pone.0290431 (PMC10599575; doi:10.1371/journal.pone.0290431)
Supplement: S1 Questionnaire — The questionnaire sheet that was used during the experiment. (PDF) [file pone.0290431.s001.pdf]

Pre-Experiment Questionnaire

Administer prior to starting experiment:

Name	Date	Subject No.

Prior Assistive Devices:

<table>
<tr><td>Do or have you used any mobility aid or other assistive technology? If so, please name them (e.g. Miniguide)</td><td colspan="6"></td></tr>
<tr><td>Which hand is your dominant hand?</td><td colspan="6"></td></tr>
<tr><td colspan="7">If the participant uses or have previously used mobility aids or other electronic assistive devices.</td></tr>
<tr><td>How frequently did you use these devices?</td><td>Multiple Times within a day</td><td>Daily</td><td>Weekly</td><td>Monthly</td><td>Yearly</td><td>Rarely or N/A</td></tr>
<tr><td>How long have you used these devices?</td><td colspan="6"></td></tr>
<tr><td colspan="7">Likert Scale</td></tr>
<tr><td>Rate these statements on how accurately they reflect your opinions.</td><td>Strongly Disagree</td><td>Disagree</td><td colspan="2">Neutral</td><td>Agree</td><td>Strongly Agree</td></tr>
<tr><td>I am dependent on the aid to navigate an environment in daily life.</td><td>1</td><td>2</td><td colspan="2">3</td><td>4</td><td>5</td></tr>
</table>

When using the aid, I feel more confident when navigating in the surroundings.	**1**	**2**	**3**	**4**	**5**
The aid allows me to easily detect and identify the objects in my surrounding environment	**1**	**2**	**3**	**4**	**5**

**Karolinska Sleepiness Scale (KSS)**	
How would you describe your current level of alertness/sleepiness.	
**1**	Extremely
**2**	Very alert
**3**	Alert
**4**	Rather Alert
**5**	Neither alert nor sleepy
**6**	Some signs of sleepiness
**7**	Sleepy, but no effort to keep awake
**8**	Sleepy, some effort to keep awake
**9**	Very Sleepy

Question for previous devices

Has it been pleasant or unpleasant to work with the product?

Unpleasant ① ② ③ ④ ⑤ ⑥ ⑦ Pleasant

How efficient was the product to use?

Inefficient ① ② ③ ④ ⑤ ⑥ ⑦ Efficient

How well did the product support the tasks you wanted to achieve?

Little support ① ② ③ ④ ⑤ ⑥ ⑦ Good support

To what degree did you feel that you were in control working with the product?

No control ① ② ③ ④ ⑤ ⑥ ⑦ Control

Was it easy or hard to learn the product?

Hard ① ② ③ ④ ⑤ ⑥ ⑦ Easy

How much physical effort is needed to operate the product?

Little effort ① ② ③ ④ ⑤ ⑥ ⑦ Much effort

How much mental effort did you experience using the product?

Little effort ① ② ③ ④ ⑤ ⑥ ⑦ Much effort

NASA Task Load Index (TLX) (21 point scale -> 7 point, score x3)

Mental Demand — How mentally demanding was the task?

0 1 2 3 4 5 6 7

Very Low — Very High

Physical Demand — How physically demanding was the task?

0 1 2 3 4 5 6 7

Very Low — Very High

Temporal Demand — How hurried or rushed was the pace of the task?

0 1 2 3 4 5 6 7

Very Low — Very High

Performance — How successful were you in accomplishing what you were asked to do?

0 1 2 3 4 5 6 7

Perfect — Failure

Effort — How hard did you have to work to accomplish your level of performance?

0 1 2 3 4 5 6 7

Very Low — Very High

Frustration — How insecure, discouraged, irritated, stressed, and annoyed wereyou?

0 1 2 3 4 5 6 7

Very Low — Very High

Mid-Experiment Questionnaire

Administer during rest break 2 of the experiment:

NASA Task Load Index (TLX) (21 point scale -> 7 point, score x3)

Mental Demand — How mentally demanding was the task?

0 1 2 3 4 5 6 7

Very Low — Very High

Physical Demand — How physically demanding was the task?

0 1 2 3 4 5 6 7

Very Low — Very High

Temporal Demand — How hurried or rushed was the pace of the task?

0 1 2 3 4 5 6 7

Very Low — Very High

Performance — How successful were you in accomplishing what you were asked to do?

0 1 2 3 4 5 6 7

Perfect — Failure

Effort — How hard did you have to work to accomplish your level of performance?

0 1 2 3 4 5 6 7

Very Low — Very High

Frustration — How insecure, discouraged, irritated, stressed, and annoyed wereyou?

0 1 2 3 4 5 6 7

Very Low — Very High

Post-Experiment Questionnaire

Administer at the end of the experiment:

NASA Task Load Index (TLX) (21 point scale -> 7 point, score x3)

Mental Demand — How mentally demanding was the task?

0 1 2 3 4 5 6 7

Very Low — Very High

Physical Demand — How physically demanding was the task?

0 1 2 3 4 5 6 7

Very Low — Very High

Temporal Demand — How hurried or rushed was the pace of the task?

0 1 2 3 4 5 6 7

Very Low — Very High

Performance — How successful were you in accomplishing what you were asked to do?

0 1 2 3 4 5 6 7

Perfect — Failure

Effort — How hard did you have to work to accomplish your level of performance?

0 1 2 3 4 5 6 7

Very Low — Very High

Frustration — How insecure, discouraged, irritated, stressed, and annoyed wereyou?

0 1 2 3 4 5 6 7

Very Low — Very High

Has it been pleasant or unpleasant to work with the product?

Unpleasant ① ② ③ ④ ⑤ ⑥ ⑦ Pleasant

How efficient was the product to use?

Inefficient ① ② ③ ④ ⑤ ⑥ ⑦ Efficient

How well did the product support the tasks you wanted to achieve?

Little support ① ② ③ ④ ⑤ ⑥ ⑦ Good support

To what degree did you feel that you were in control working with the product?

No control ① ② ③ ④ ⑤ ⑥ ⑦ Control

Was it easy or hard to learn the product?

Hard ① ② ③ ④ ⑤ ⑥ ⑦ Easy

How much physical effort is needed to operate the product?

Little effort ① ② ③ ④ ⑤ ⑥ ⑦ Much effort

How much mental effort did you experience using the product?

Little effort ① ② ③ ④ ⑤ ⑥ ⑦ Much effort

Trial Runsheet

Please fill during the experiment to track trials:

-(✓) Tick item successfully found the target

-(**X**) Cross the item if the participant reached this item but it was not correct, tick the intended target.

-(**+**) if participant stated they heard the item in AR Condition

-(**-**) if participants stated they did not hear the item in AR Condition.

-(?) if participants stated an item that wasn’t part of the AR condition

-Co= Control, CL=Clock, AR=FAD

Trial	Item				Notes:			
	Book	Bottle	Bowl	Cup	Conditions			
1					CO	CL	AR	
2					CO	CL	AR	
3					CO	CL	AR	
4					CO	CL	AR	
5					CO	CL	AR	
6					CO	CL	AR	
7					CO	CL	AR	
8					CO	CL	AR	
9					CO	CL	AR	
10					CO	CL	AR	
11					CO	CL	AR	
12					CO	CL	AR	
13					CO	CL	AR	
14					CO	CL	AR	
15					CO	CL	AR	
16					CO	CL	AR	
17					CO	CL	AR	
18					CO	CL	AR	
19					CO	CL	AR	
20					CO	CL	AR	
Rest Break								
21					CO	CL	AR	

	Book	Bottle	Bowl	Cup				
22					CO	CL	AR	
23					CO	CL	AR	
24					CO	CL	AR	
25					CO	CL	AR	
26					CO	CL	AR	
27					CO	CL	AR	
28					CO	CL	AR	
29					CO	CL	AR	
30					CO	CL	AR	
31					CO	CL	AR	
32					CO	CL	AR	
33					CO	CL	AR	
34					CO	CL	AR	
35					CO	CL	AR	
36					CO	CL	AR	
37					CO	CL	AR	
38					CO	CL	AR	
39					CO	CL	AR	
40					CO	CL	AR	
Rest Break								
41					CO	CL	AR	
42					CO	CL	AR	
43					CO	CL	AR	
44					CO	CL	AR	
45					CO	CL	AR	
46					CO	CL	AR	
47					CO	CL	AR	
48					CO	CL	AR	
49					CO	CL	AR	
50					CO	CL	AR	
51					CO	CL	AR	
52					CO	CL	AR	
53					CO	CL	AR	

	Book	Bottle	Bowl	Cup				
**54**					**CO**	**CL**	**AR**	
**55**					**CO**	**CL**	**AR**	
**56**					**CO**	**CL**	**AR**	
**57**					**CO**	**CL**	**AR**	
**58**					**CO**	**CL**	**AR**	
**59**					**CO**	**CL**	**AR**	
**60**					**CO**	**CL**	**AR**	
**End**								

Trial Runsheet

	Book	Bottle	Bowl	Cup	Find item?	No. Reache s	Comments ?
**1**							
**2**							
**3**							
**End**							
